# Impact of Ultrasound-Treated Emulsion Gels on the Structure of Purees for Oropharyngeal Dysphagia

**DOI:** 10.3390/molecules30193933

**Published:** 2025-10-01

**Authors:** Minfang Luo, Winifred Akoetey, Nuria Martí, Domingo Saura, Farah Hosseinian

**Affiliations:** 1 Food Science, Chemistry Department, Carleton University, Ottawa, ON K1S 5B6, Canada; 2 Health Biotechnology of Elche, Miguel Hernández University, 03202 Elche, Alicante, Spain; 3 Institute of Biochemistry, Carleton University, Ottawa, ON K1S 5B6, Canada

**Keywords:** emulsion gel, psyllium husk, inulin, ultrasound, carbohydrate-hydrolyzing enzymes, oropharyngeal dysphagia

## Abstract

This study investigated the effects of inulin concentration and ultrasonic homogenization on the particle size distribution and microstructure of oil-in-water emulsion gels stabilized with psyllium husk. These gels were then incorporated into meal purees formulated for individuals with dysphagia. Under ultrasound treatment, an increase in inulin from 0% to 20% reduced the average droplet size from 14.98 μm to 1.58 μm, indicating a synergistic effect between ultrasound treatment and inulin in reducing and stabilizing droplet size. The optimal formulation under ultrasound was 20% (*w*/*w*) inulin. Scanning electron and polarized light microscopy confirmed that ultrasonic homogenization improved emulsion integrity by minimizing droplet size and promoting encapsulation. Inulin-rich emulsion gels, when added to purees, reduced structural voids, improved matrix cohesion, and lowered expressible fluid content. Enzymatic assays showed enhanced inhibition of α-amylase and α-glucosidase, indicating increased resistance to oral enzymatic degradation. Importantly, substituting emulsion gels at 10% (*w*/*w*) did not compromise puree firmness. All formulations met International Dysphagia Diet Standardization Initiative (IDDSI) Level 4 requirements, confirming their suitability for individuals with oropharyngeal dysphagia (OD). These findings demonstrate the potential of psyllium husk-stabilized emulsion gels as innovative texture-modifying agents for dysphagia-friendly food development.

## 1. Introduction

Dysphagia, characterized by difficulty in swallowing, affects approximately 35% of older adults in Canada and up to 50% of those who are hospitalized [1,2]. This condition significantly impacts nutrition, hydration, medication intake, and overall quality of life, and is associated with serious complications, including aspiration, malnutrition, and increased morbidity. Standard clinical management of dysphagia includes the use of thickened liquids and texture-modified foods to reduce the risk of aspiration and facilitate safe swallowing [3]. Pureed foods must be smooth, cohesive, and require minimal oral manipulation. Thickeners, particularly starch-based ones, are often used to improve rheological properties; however, their efficacy is limited by enzymatic degradation. Salivary α-amylase rapidly hydrolyzes starch, resulting in viscosity loss during oral processing and compromising bolus stability [4,5].

Adding gums (e.g., xanthan, guar, carrageenan) to modify food texture for dysphagia can be problematic, as they primarily increase viscosity without necessarily improving swallowing safety. Effective dysphagia-friendly textures require not just thickness, but also bolus cohesiveness, lubrication, and controlled breakdown—qualities gums alone may not provide. In addition to mechanical safety, dysphagia diets frequently lack sufficient dietary fibre due to high levels of food processing [6,7]. Fibre deficiency in this population may contribute to gastrointestinal discomfort, constipation, and poor cardiometabolic outcomes, particularly among individuals with reduced food intake due to age-related changes or comorbid conditions. Although psyllium husk has no RDA, the Adequate Intake (AI) for total dietary fibre is 14 g per 1000 kcal (≈25 g/day for women; 38 g/day for men), and for psyllium specifically, about 7 g/day of soluble fibre is supported for lowering LDL-cholesterol [8,9].

To address these challenges, emulsion gels represent a promising dual-function solution, providing structural modification and delivering dietary fibre. These semi-solid systems entrap oil droplets in a gel matrix formed by hydrophilic polymers, improving texture, reducing syneresis, and enhancing nutrient encapsulation [10,11]. Psyllium husk (Plantago ovata), a mucilaginous fibre with strong water-binding and gel-forming capacity, offers both thickening and enzyme inhibitory properties [12,13]. However, its rapid gelation complicates uniform incorporation into foods [14]. Forming a gel system with the psyllium husk before incorporation into foods would overcome the possible limitations of food uniformity. In addition, the inclusion of a secondary fibre source, inulin, a soluble dietary fibre with prebiotic effects and fat-mimicking texture, could act as a co-stabilizer to improve uniformity, stability, and nutritional value [15,16].

The primary objectives of this study were to develop and optimize food-grade psyllium husk-based emulsion gels and to evaluate their applicability in commercial puree foods intended for individuals with dysphagia. The study involved the following: (a) Characterizing the gel microstructure using polarized light microscopy, cryo-scanning electron microscopy (cryo-SEM), and particle size analysis; (b) Assessing functional properties, including total expressible fluid and inhibitory activity against α-amylase and α-glucosidase; and (c) Evaluating the modified puree formulations (containing 10% *w*/*w* emulsion gel) using the International Dysphagia Diet Standardization Initiative (IDDSI) framework to determine their suitability as Level 4 puree foods. These findings offer a foundation for the development of safer, more nutritious dysphagia-friendly foods.

## 2. Results and Discussion

### 2.1. Structural and Stability Analysis of Emulsion Gels

Structural characterization across inulin levels under ultrasound was conducted using polarized light microscopy (PLM) for visualization of overall droplet morphology and qualitative dispersion, particle-size analysis for quantification of the mean droplet size and distribution, and cryo-scanning electron microscopy (cryo-SEM) to examine the droplet–matrix interfaces.

#### 2.1.1. Polarized Light Microscopic Observation

**Figure 1:**
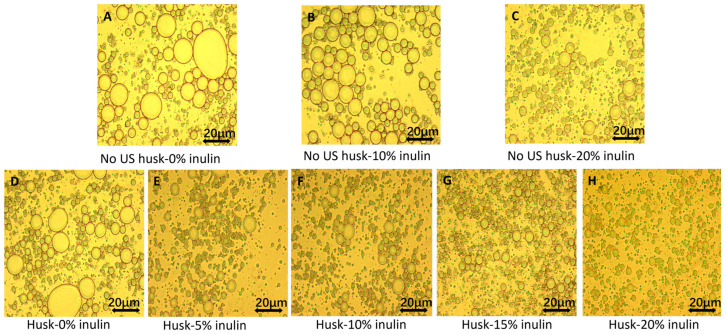
PLM observation (500×) of ultrasound-treated (US) and untreated (No-US) 3% psyllium husk emulsion gels at different inulin concentrations. Panels (**A**–**C**): No-US (0%, 10%, 20% inulin). Panels (**D**–**H**): US (0%, 5%, 10%, 15%, 20% inulin). Scale bar = 20 μm.

PLM images (Figure 1) showed that inulin concentration modulated the qualitative droplet morphology of 3% psyllium husk emulsion gels. In Figure 1A–C (no ultrasound; 0%, 10%, 20% inulin), droplets appeared larger and more polydisperse; with increasing inulin, droplets became visually smaller and more uniformly dispersed. This trend aligns with reports that higher inulin is associated with smaller droplets and improved stability [17,18]. For the same inulin level (A vs. D; B vs. F; C vs. H), no-ultrasound images displayed larger droplets than their ultrasound-treated counterparts. Reduction in droplet size by ultrasound can be attributed to the shear force generated by the cavitation of the ultrasonic wave, breaking droplets into smaller sizes [19]. At a lower inulin concentration, the micrographs suggested droplet flocculation of larger droplets within the psyllium matrix. In emulsion-filled gels, immobilized droplets can act as fillers; limited, immobilized clustering may contribute to small-strain rigidity when interfaces are well covered, whereas extensive flocculation of poorly covered, larger droplets disrupts the network and increases coalescence risk [20,21,22]. In the present system, the combination of ultrasound with higher inulin produced smaller, well-stabilized droplets and greater spacing between droplets, which are consistent with improved stability. These qualitative trends are corroborated by particle size distributions (Section 2.1.2) and cryo-SEM (Section 2.1.3).

#### 2.1.2. Particle Size Distribution

**Figure 2:**
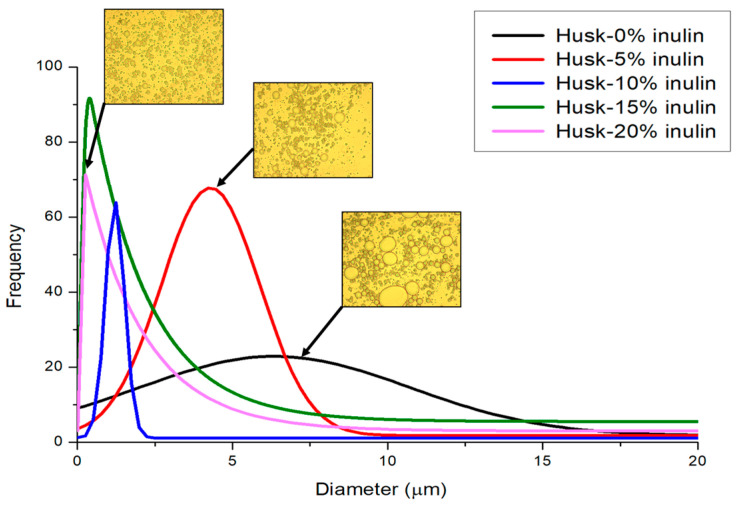
Particle size analysis of ultrasound-treated 3% psyllium husk emulsion gel with different concentrations of inulin.

Particle size distributions (Figure 2) were consistent with the PLM observations. At 0% inulin (under ultrasound), the distribution was broad, indicating wide variability in droplet size. As inulin was increased from 0% to 20% under ultrasound, the average droplet size decreased from 14.98 µm to 1.58 µm, and the distributions became narrower (sharper peaks), indicating a more uniform droplet population. The narrower distributions at higher inulin concentration reduce the possibility of forming large and flocculated clusters, which supports matrix integrity. These results provide quantitative evidence of stabilization associated with inulin under ultrasound [17,18,23].

#### 2.1.3. Cryo-Scanning Electron Microscopic Observation

**Figure 3:**
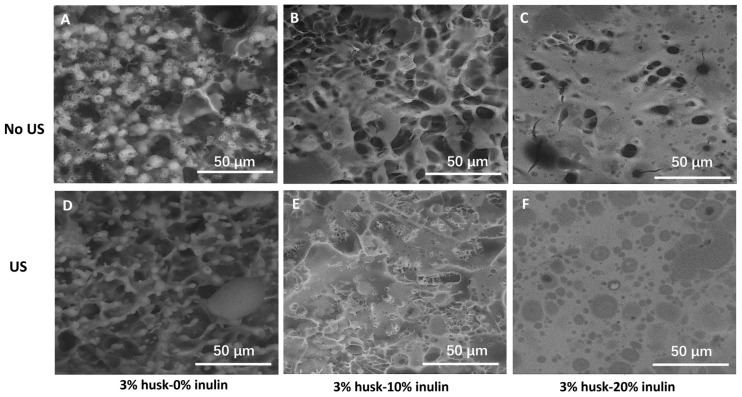
SEM micrographs of 3% psyllium husk emulsion gels at different inulin levels with and without ultrasound (US). Panels (**A**–**C**): No-US, 0%, 10%, 20% inulin (respectively). Panels (**D**–**F**): US, 0%, 10%, 20% inulin (respectively). Scale bar = 50 µm in all panels.

The SEM images (Figure 3) revealed that inulin addition and ultrasonic treatment affected the morphology of the husk emulsion gel system. In Figure 3A–C (no ultrasound; 0–20% inulin), the microstructure at 0% inulin appeared heterogeneous and porous, and droplet surfaces became progressively smoother with fewer cavities as inulin increased, suggesting that inulin was in a packed arrangement at the droplet interface and could also form an interfacial layer coating the surface of oil droplets. A similar result was observed for an emulsion gel developed with inulin/konjac glucomannan complex [17]. In that study, they reported that the polysaccharide complex was adsorbed at the oil–water interface, and that the complex also created a mechanical barrier to prevent interfacial film rupture. Polymer adsorption at the interface can enhance the mechanical properties of the interfacial films and connections between the interface and the matrix gel, which can immobilize oil droplets and inhibit coalescence; therefore, larger droplets have weaker interfacial films and are more prone to coalescence [24,25].

The SEM image of the 20% inulin emulsion gel showed a more uniformly packed emulsion gel with fewer cavities compared to the other concentrations, indicative of a stable gel network. These findings are consistent with Lu et al. (2021), who reported that emulsion gels with unevenly distributed larger pores were associated with incomplete coverage of oil droplets by the gel network, resulting in a weak and loose gel structure [25].

For the same inulin levels (A vs. D; B vs. E; C vs. F), ultrasound-treated emulsion gel (Figure 3D–F) showed higher apparent integrity of the interfacial coating and smoother droplet surfaces than the no-ultrasound-treated emulsion gel (Figure 3A–C). At 20% inulin, the dark area in the no-ultrasound-treated emulsion gel (Figure 3C) showed that droplets were not completely covered by the inulin layer. However, the surface of the ultrasound-treated emulsion gel (Figure 3D) was smooth, without a gap, suggesting that the droplet is fully covered by the inulin layer at that concentration. Similarly, Xiong et al. (2019) reported that more emulsifier molecules were adsorbed on the surface of the oil droplets in ultrasound-treated xanthan gum emulsion compared to the untreated emulsion [26]. The increased particle adsorption on the surface of the emulsion droplet by ultrasound occurs due to momentum transfer from the fluid to the particles and droplets caused by random cavitation events. The momentum transfer can overcome the stabilizing energy barriers that normally prohibit nanoparticles from spontaneously adhering to the oil–water interface [27].

The microstructural evidence from the tests conducted indicates that a psyllium-based hydrogel network forms the continuous, water-binding phase, while oil droplets, interfacially stabilized (with inulin) and reduced in size by ultrasound, are embedded and immobilized within this matrix, yielding an emulsion-filled gel. At a higher inulin concentration, there is a denser, more continuous matrix with smoother droplet interfaces, consistent with improved network integrity. Inulin reduced droplet size, flocculation, and improved interfacial coverage; therefore, the droplets were effectively immobilized within the psyllium network [20]. Beyond interfacial effects, inulin may also reinforce the continuous phase via the formation of junctions, which align with the denser matrix observed [28]. This hybrid structure provides the lubrication–cohesion balance relevant to swallowing. Deformable droplets can enter the tongue–palate, contact and roll/slide, lowering friction [29], while the gel matrix supplies yield stress and cohesive bolus control [4,30]. The resulting structure is consistent with emulsion-gel design principles [21,31].

### 2.2. Effect of Emulsion Gel Addition in Meat Puree

#### 2.2.1. Microstructure Morphology

**Figure 4:**
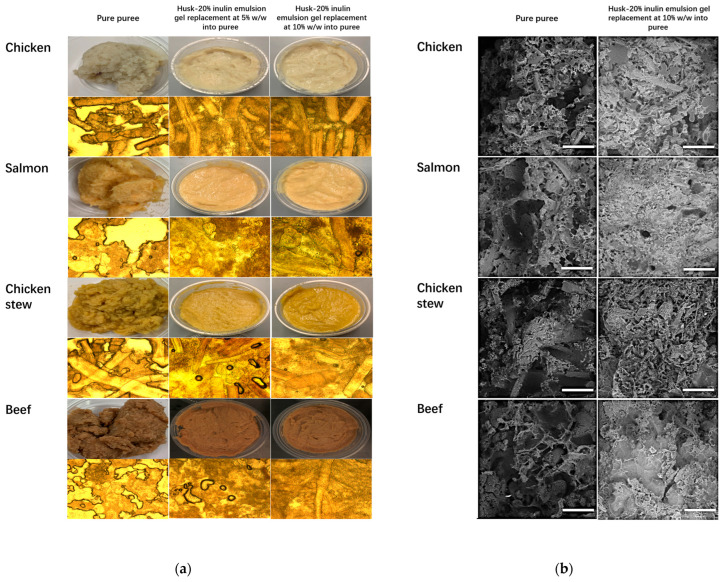
(**a**) PLM and photo images of puree samples incorporated with the husk–20% inulin emulsion gel at 5% *w*/*w* and 10% *w*/*w* in comparison to the pure puree sample. (**b**) Cryo-SEM imaging of puree samples before and after incorporation of husk–20% inulin emulsion gel at 10% *w*/*w*. Scale bar = 50 μm.

The effect of emulsion gel addition on the microstructure of meat puree is shown in the PLM image (Figure 4a) and the Cryo-SEM image (Figure 4b).

The morphology of the control purees in the PLM images (Figure 4a) shows large voids between sample particles. These voids become less evident with increased addition of the emulsion gel (0–10% *w*/*w*). At 10% inclusion, no voids are observed. Similarly, in the Cryo-SEM images (Figure 4b), all pure puree samples exhibited the formation of different-sized cavities creating structures with a rough and porous appearance. The high proportion of cavities can be interpreted as water, oil, or lipid, and air expansion in the protein network [16]. The 10% emulsion gel-incorporated puree had fewer cavities and a more compact structure compared to pure puree because the emulsion gel contained a high amount of inulin [32]. The soluble fraction from dietary fibre can distribute evenly in the meat matrix; hence, it has a higher opportunity to interact with the medium. This interaction contributes to the development of higher water and fat binding properties, resulting in a more homogeneous meat matrix [32,33]. According to the study from Morin et al. (2004), a finely chopped meat system is structurally similar to an emulsion, where fat globules (the dispersed phase) remain intact and are suspended in a protein–water solution (the continuous phase) [34]. Additionally, it has been reported that meat matrices with homogeneous and compact structures, as observed in the puree sample with 10% emulsion gel (Figure 4), are classified as strong meat emulsions with good water and fat binding properties, while those with irregular and less compact structures, as seen in the pure puree samples (Figure 4), are classified as poor meat emulsions with weak binding properties [35].

Emulsion gels are semi-solid systems formed by gelation of the continuous phase of an emulsion or by droplet aggregation via hydrophilic polymers. These systems trap oil droplets within a viscoelastic matrix, leading to enhanced texture, reduced syneresis, and better stability of encapsulated nutrients [10,11]. They are particularly suited for dysphagia foods where moistness, uniformity, and structural cohesion are essential to prevent choking and aspiration [36]. By contrast, gums such as xanthan thicken by binding water and may reduce polyphenol bioaccessibility through hydrogen and hydrophobic interactions. Emulsion gels, however, can both modify texture and protect polyphenols, controlling their release and enhancing stability and bioavailability in texture-modified foods [36,37].

#### 2.2.2. Total Expressible Fluid (TEF)

**Figure 5:**
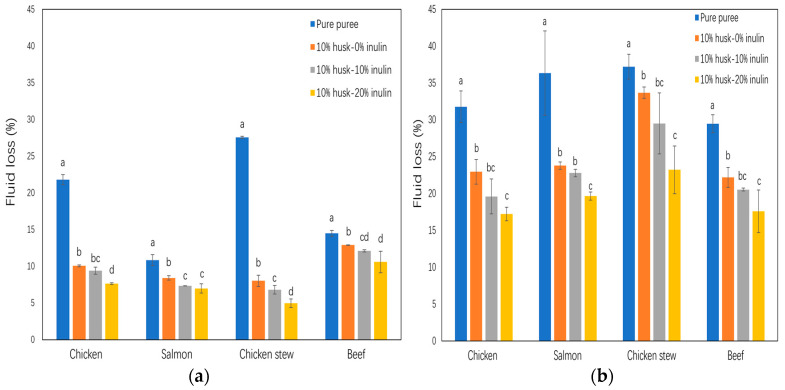
Effect of incorporation of 10% psyllium husk emulsion gel with 0%, 10%, and 20% inulin in puree samples on fluid loss (**a**) at 70 °C water bath for 30 min, and (**b**) at microwave oven heating with 700 W power for 2 min. Different letters represent significant differences at *p* < 0.05.

Minimizing fluid separation in dysphagia food is essential for the development of safe dysphagia-friendly food products that effectively reduce the risk of choking and aspirational pneumonia in individuals with dysphagia [38]. Figure 5a and Figure 5b represent the results of TEF of different purees by water bath and microwave oven heating, respectively.

TEF of water bath-heated or microwave-heated pure puree (chicken, salmon, chicken stew, and beef) was significantly higher than that of puree with 10% *w*/*w* husk—0% inulin emulsion gel incorporation. Water bath and microwave oven heating methods were selected in this study because they are common household and institutional heating methods. Increased inulin concentration in emulsion gel (from 0% to 20%) incorporation in the purees further reduced their TEF. So, the puree with husk—20% inulin emulsion gel had the lowest TEF within the same puree group. For example, within the chicken puree group (Figure 5a), the pure puree (no emulsion gel incorporation) had the highest TEF (31.77% mean value), while the puree with 10% *w*/*w* replaced by husk—20% inulin emulsion gel had the lowest TEF (17.22% mean value). The microwave-heating method (Figure 5b) yielded a similar result; the puree with husk—20% inulin emulsion gel incorporation had the lowest TEF within the same puree group.

TEF reduction with inclusion of the husk emulsion gel is attributed to the water-binding and immobilizing capacity of the psyllium network, together with non-covalent associations between the psyllium arabinoxylan and proteins in the puree matrix. For example, hydrogen bonding and electrostatic interactions have been reported to reinforce protein gels or composite protein–polysaccharide matrices, and improve water retention in myofibrillar protein systems [39,40]. In parallel, inulin increases continuous-phase solids and contributes to water/fat retention, further lowering expressible fluid [41]. These network-level and interfacial effects collectively strengthen the three-dimensional matrix and reduce fluid separation during heating. Psyllium husk provides both soluble and insoluble fibre, of which about 80% is soluble and mucilaginous, enabling gel formation and high-water binding [11,12]. These gelling and water-binding properties, combined with the inulin-associated retention noted above, are consistent with the observed TEF reductions [41,42].

In comparison to water bath-heated samples, microwave heating resulted in higher TEF, similar to results by Chang et al. (2011), who reported significantly greater cooking loss and higher insoluble collagen content in microwave-heated beef semitendinosus muscle [43]. The increased insoluble collagen suggests insufficient gelatinization of collagen, which normally forms a gel network capable of retaining water during heating [43]. The increased insoluble collagen, combined with the structural disruption caused by rapid and uneven microwave heating, leads to greater moisture loss from the meat tissue [43]. Additionally, microwave heating creates a higher temperature in the centre of the puree, and the high steam pressure drives more water from within the sample to the surface [44].

#### 2.2.3. Effect of Emulsion Gel Addition on the Firmness of Puree Samples

**Figure 6:**
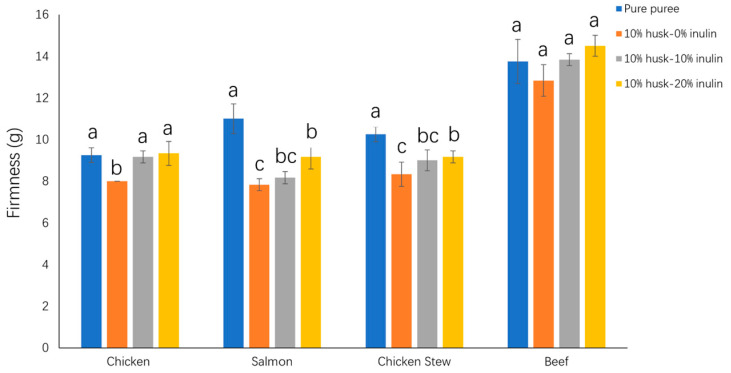
Effect of incorporation of 10% *w*/*w* psyllium husk emulsion gel with 0%, 10%, and 20% inulin in puree samples on the firmness of puree samples. Different letters represent significant differences at *p* < 0.05.

The firmness of food for dysphagia patients is also an important factor to consider, as a harder texture requires more time and energy for oral processing. This increased effort during consumption can lead to fatigue and discomfort for individuals with dysphagia [38]. As shown in Figure 6, when husk—0% inulin emulsion gel was incorporated into 10% *w*/*w* puree, all purees except beef puree had significantly (*p* < 0.05) lower firmness values than the pure puree, indicating that the incorporation of husk—0% inulin emulsion gel makes the puree softer. Grigelmo-Miguel et al. (1999) and López-López et al. (2010) also reported that the incorporation of insoluble fibre could decrease the firmness of cooked meat [33,45]. The reduction in firmness may be due to the effect of a three-dimensional network of the insoluble dietary fibre within the meat system that could disrupt the protein-water or protein-protein gel network, resulting in a reduced meat gel strength and a softer texture [33,45,46].

It was observed that the purees with husk—10% inulin emulsion gel had significantly (*p* < 0.05) higher firmness values compared to those with husk—0% inulin emulsion gel. In addition, purees with husk—20% inulin emulsion gel incorporation had higher values of firmness compared to those with husk—10% inulin emulsion gel and reached similar (*p* > 0.05) values of firmness with the control puree. This indicates that the addition of inulin is useful to compensate for the changes in texture due to adding husk emulsion gel alone in the meat puree. Similarly, Paglarini et al. (2022) demonstrated that the addition of inulin emulsion gel could increase the water and oil holding capacity in cooked sausages, resulting in higher firmness of the sausage [16]. At higher inulin concentrations, more water is absorbed and incorporated into a tightly packed crystalline gel network. This structure reduces the extensibility of the polymer chains due to the high density of intermolecular bonds, thereby enhancing the physical stability of the emulsion, and the creaming phenomenon is effectively inhibited [47].

Interestingly, pure beef puree had the highest firmness among all pure purees, but no significant differences were observed for its firmness after incorporation of emulsion gels. It might be because the strength of pure beef puree was too strong to be affected by the addition of husk emulsion gel.

### 2.3. Enzyme Inhibition

#### 2.3.1. Alpha-Amylase Inhibition

Most texture-modified foods for patients with dysphagia utilize starch as a thickening agent. However, these foods tend to be rapidly hydrolyzed by α-amylase in the mouth into amylose and amylopectin, thus significantly reducing viscosity during mastication [4,5]. This enzymatic breakdown compromises bolus stability and can increase the risk of aspiration. Therefore, selecting thickeners that resist enzymatic degradation by α-amylase is crucial in developing safer dysphagia-friendly foods.

**Figure 7:**
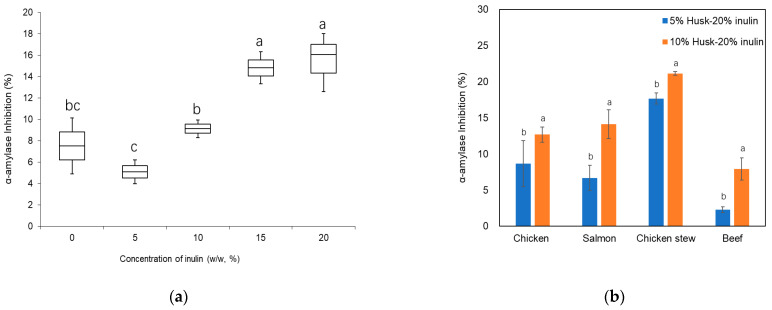
Inhibitory potency of (**a**) psyllium husk emulsion gel in the presence of different concentrations of inulin at 200× dilution, and (**b**) puree samples with incorporated 5% or 10% husk–20% inulin emulsion gel against α-amylase (1 U/mL) activity at 1% starch concentration. Different letters represent significant differences at *p* < 0.05.

Figure 7a shows the α-amylase inhibitory effect of husk-emulsion gel with 0, 5, 10, 15, and 20% *w*/*w* inulin addition at 5 mg/mL. Inhibitory activity generally increased with increasing inulin concentration from 0% to 15%. However, inhibition at 0–10% were not significantly different. The inhibition of α-amylase by husk- 0% inulin emulsion gel is in agreement with findings of Ahmed & Urooj (2010), who reported that dietary fibres like psyllium husk and wheat bran can inhibit amylase activity by binding glucose [13]. Moreover, dietary fibre can enhance the entanglement and overlap with molecular chains of starch via noncovalent bonds, resulting in improved microstructure compactness of the starch and reduced accessibility to the α-amylase [48,49]. The α-amylase inhibitory activity of the emulsion gel at 20% inulin was not significantly different from that of 15% inulin concentration. The inhibitory activity of husk-emulsion gel with 15% and 20% inulin at 5 mg/mL on α-amylase was 14.82 ± 2.11% and 16.06 ± 3.03%, respectively.

The pure puree samples (salmon, chicken, chicken stew, or beef) were used as the control group in Figure 7b. The α-amylase inhibitory activity of puree samples containing 5% *w*/*w* and 10% *w*/*w* husk—20% inulin emulsion gel (Figure 7b) demonstrated a greater ability to delay α-amylase activity compared to the pure puree samples. The inhibitory activity of puree samples with 10% *w*/*w* replacement by the emulsion gel was higher than that of puree with 5% *w*/*w* replacement. Amongst the purees with 10% *w*/*w* replacement by husk—20% inulin emulsion gel, the chicken stew had the highest amylase inhibition (21.14%), followed by salmon and chicken (14.12 and 12.68% inhibition, respectively). The beef had the lowest amylase inhibition of 7.93%. Guo et al. (2020) reported that the hydroxyl (-OH) and carboxyl groups (-COOH) on the polysaccharides’ branched chain can interact with the amino acid residues of digestive enzymes (α-amylase and α-glucosidase), thus inhibiting their activity [50]. Overall, under these screening conditions, our results indicate that the incorporation of husk—20% inulin emulsion gel will be beneficial in delaying dietary starch breakdown in food products and thereby maintaining their consistency with the addition of starch-based thickener during oral digestion.

#### 2.3.2. Alpha-Glucosidase Inhibition

**Figure 8:**
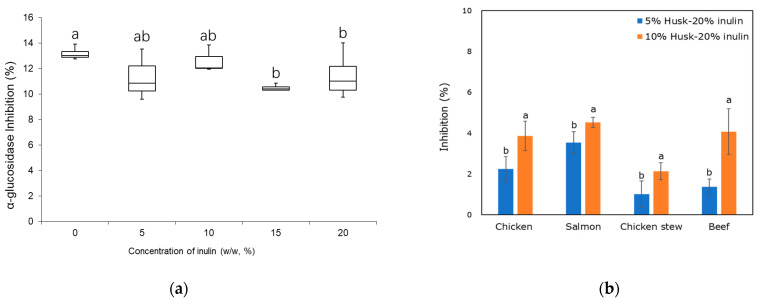
Inhibitory potency of (**a**) psyllium husk emulsion gel in the presence of different concentrations of inulin at 200× dilution, and (**b**) puree samples with incorporated 5% or 10% husk–20% inulin emulsion gel against α-glucosidase (1 U/mL) activity at 1 mM PNPG concentration. Different letters represent significant differences at *p* < 0.05.

α-glucosidase inhibition is a treatment option for patients with diabetes. The enzyme breaks down oligosaccharides and disaccharides into glucose for absorption in the small intestine. The delay for the hydrolyses is important in regulating the amount of glucose that enters the bloodstream [51]. Modified foods with this delay could be beneficial for patients with diabetes as well. In this study, all emulsion gel samples delayed α-glucosidase activity (Figure 8a); however, inhibitory activity decreased slightly with increasing inulin concentration from 0% to 20%. The husk emulsion gel without inulin had the highest α-glucosidase inhibition (12.93 ± 0.19%), while the husk-emulsion gel with 20% *w*/*w* inulin addition had the lowest glucosidase inhibition of (10.6 ± 0.91%). This inhibitory effect may be primarily attributed to the psyllium fibre, which could reduce enzyme mobility and promote adhesion to starch granules, thereby interfering with enzyme–substrate interactions [52].

All puree samples, with either 5% or 10% *w*/*w* replacement of the emulsion gel, delayed α-glucosidase activity (Figure 8b). Specifically, the inhibitory activity of puree samples with 10% *w*/*w* replacement of the emulsion gel was higher than that with 5% *w*/*w* replacement, indicating that the addition of a higher amount of the emulsion gel led to a greater α-glucosidase inhibitory effect. Among the purees with 10% *w*/*w* replacement with husk—20% inulin emulsion gel, salmon had the highest glucosidase inhibition (4.54%), followed by beef and chicken (4.08 and 3.87%, respectively). The chicken stew had the lowest glucosidase inhibition of 2.13%. Previous studies also suggested that psyllium mucilage could delay α-glucosidase activity and entrap glucose in vitro and in vivo [53,54], but this mechanism has not been explored. Overall, under these screening conditions, it can be concluded that husk—20% inulin emulsion gel incorporation in food products will beneficially delay α-glucosidase activity.

### 2.4. International Dysphagia Diet Standardization Initiative (IDDSI) Tests

**Figure 9:**
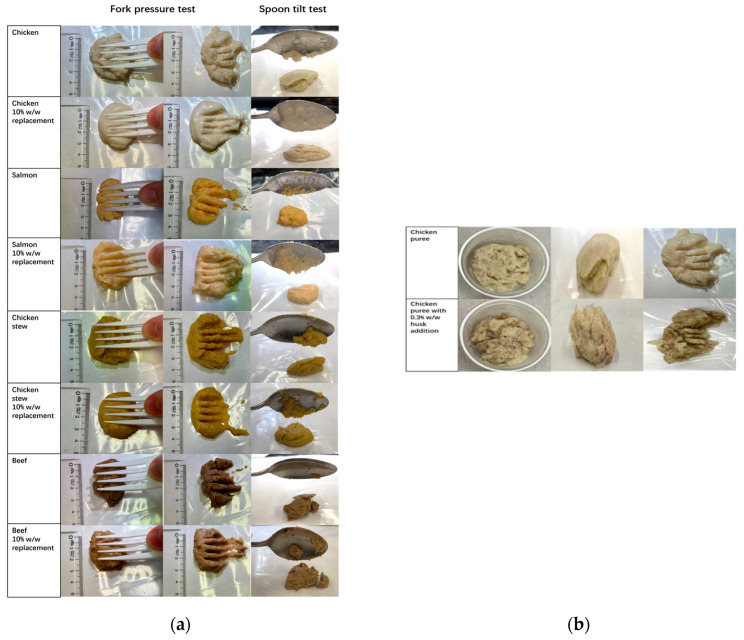
(**a**) IDDSI tests: Fork pressure test and spoon tilt test on pure puree samples and puree samples with incorporated 10% *w*/*w* husk–20% inulin emulsion gel, and (**b**) visual comparison of direct husk addition: chicken puree without and with 0.3% *w*/*w* directly added psyllium husk.

A critical requirement in dysphagia food formulation is ensuring that texture-modified foods meet the standards set by the International Dysphagia Diet Standardization Initiative (IDDSI). To evaluate the suitability of puree samples containing husk–inulin emulsion gel, two standard IDDSI Level 4 tests—the fork pressure test and the spoon tilt test—were conducted across various food matrices (Figure 9a).

The fork pressure test confirmed that all samples, including chicken, salmon, chicken stew, and beef with 10% *w*/*w* emulsion gel, could be easily mashed with a fork into particles no larger than 4 mm. Distinct indentation marks remained after compression, satisfying the IDDSI requirement for puree foods. Similarly, in the spoon tilt test, all samples maintained their shape on the spoon and slid off cleanly with minimal residue, indicating sufficient cohesiveness and moisture content. These results demonstrate that the incorporation of husk–inulin emulsion gel does not compromise the structural integrity of puree foods and that all formulations meet IDDSI Level 4 standards. Consistent with IDDSI practice, preparations were presented as emulsion gels in which psyllium was pre-hydrated during processing and homogeneously dispersed within an IDDSI Level 4 matrix (spoon-thick, cohesive, no separate thin liquid) [55,56]. According to the IDDSI classification, IDDSI Level 4 texture is suitable for individuals who experience pain during swallowing and chewing, are edentulous, or have unsuitable dentures [55]. These individuals require liquids to be thickened to the extent that they can be consumed with a spoon rather than through a straw; therefore, consumption of IDDSI Level 4 products can enhance safety by reducing the risk of liquid aspiration and lowering bolus velocity in patients with dysphagia [56].

While these findings affirm the effectiveness of the emulsion gel as a thickening and stabilizing agent, a supplementary visual comparison was conducted to assess the limitations of using psyllium husk in its raw form. As shown in Figure 9b, chicken puree with 0.3% *w*/*w* directly added psyllium husk displayed a visibly coarse, irregular texture with poorly dispersed fibre particles. This contrasted sharply with the plain chicken puree, which appeared smoother and more cohesive. The inconsistent distribution and textural roughness of the direct husk-added sample indicate potential challenges in both mouthfeel and bolus safety for individuals with dysphagia.

These observations highlight the importance of delivery format when incorporating functional fibres like psyllium into dysphagia-friendly foods. Direct addition may lead to nonhomogeneous textures and compromised swallowing safety, whereas pre-formulating psyllium husk into an emulsion gel matrix ensures uniform dispersion and improved water retention. Moreover, the emulsion gel serves a dual purpose by improving the texture of the puree food while simultaneously increasing its dietary fibre content, helping to address the common issue of fibre deficiency in dysphagia diets.

In addition to safety concerns, nutritional inadequacy, particularly low dietary fibre intake, is a major issue in dysphagia management [7]. Institutional pureed diets are often highly processed and low in whole food content, leading to insufficient fibre intake [6]. A fibre-deficient diet can contribute to gastrointestinal discomfort, constipation, and cardiovascular diseases [57]. These effects are especially concerning for vulnerable populations with dysphagia, who may already have reduced food intake due to chewing difficulties, low appetite, or comorbidities such as cognitive impairment. Incorporating functional fibres such as inulin into texture-modified foods may help address these concerns. Inulin is a soluble dietary fibre known for its smooth, creamy texture and ability to mimic fat in food matrices [15,16]. Moreover, inulin resists digestion in the upper gastrointestinal tract and is fermented by colonic microbiota to produce short-chain fatty acids, which promote the growth of beneficial bacteria such as Bifidobacteria and Lactobacilli, enhance calcium absorption, and support immune function [15]. Hence, delivering dietary fibre through thickened or pureed products offers a practical and targeted approach to improving gut health and nutritional adequacy in individuals with dysphagia.

## 3. Materials and Methods

### 3.1. Materials

Sunflower oil was from Saporito Foods (Montreal, QC, Canada). Inulin was extracted from Jerusalem artichoke by Nutia Food Ingredients (Kentwood, MI, USA). Sodium alginate (Landor Trading Company) was purchased via Amazon. Psyllium husk was purchased from a local grocery store (Bulkbarn.ca). Sodium carbonate was purchased from VWR International Co. (Mississauga, ON, Canada). Tween 80 (polyoxyethylene-20-sorbitan monooleate) was purchased from Fisher Scientific (Ottawa, ON, Canada). Alpha-glucosidase, α-amylase, P-NPG (p-Nitrophenyl-α-D-glucopyranoside), 3,5-Dinitrosalicylic acid, and potassium sodium tartrate tetrahydrate were purchased from Sigma-Aldrich (St. Louis, MO, USA). Salmon puree, chicken puree, chicken stew puree, and beef puree were kindly provided by Apetito HFS Ltd. (Orléans, ON, Canada). The purees contained xanthan gum as a thickener.

### 3.2. Formulation and Preparation of Psyllium Husk Emulsion Gels

Based on experimental unpublished data, a 3% psyllium husk gel was found to be the most suitable for use as an emulsion gel. The emulsion gel was prepared as follows.

Psyllium husk (1.5 g) was first dissolved completely in 28.7 g of water (80 °C), then cooled to room temperature (23 °C). Next, 10 g of alginate solution (0.5 g sodium alginate in 9.5 g water) and sunflower oil (8.8 g) were added to the husk solution, followed by mechanical stirring using a Tissuemiser (Fisher Scientific, Ottawa, ON, Canada) until the emulsion gel appeared homogenous.

Emulsion gels containing inulin were prepared with varying amounts of inulin (0, 2.5, 5, 7.5, or 10 g) to achieve target concentrations of 0%, 5%, 10%, and 20%, respectively. The inulin was added during emulsion preparation by replacing an equivalent amount of water in the husk solution.

### 3.3. Ultrasound-Treated Emulsion Gel

Ultrasound was applied to prepare an emulsion gel with the above formulation (Section 3.2) based on the method of Leong et al. (2017), with modifications [19]. Ultrasound processing was carried out using UIP500hd (Hielscher, Germany) at 20 kHz fitted with a titanium horn (tip size 18 mm). Psyllium husk (1.5 g) was first dissolved completely in 28.7 g of water (80 °C), then cooled to room temperature (23 °C). To achieve target inulin concentrations of 0%, 5%, 10%, and 20%, inulin powder at 0, 2.5, 5, 7.5, and 10 g was added, respectively, to the husk solution and mixed by ultrasound at 20 W power for ~10–20 s until completely dissolved. The equivalent amount of water in the husk solution was replaced with the inulin during the emulsion gel preparation. Sunflower oil (8.8 g) was then mixed with the aqueous phase by ultrasound at 100 W power for ~60–120 s. The mixture was then remixed with 10 g of alginate solution (0.5 g sodium alginate in 9.5 g water) at 100 W until the emulsion appeared homogenous. The formulated emulsions were stored at 4 °C for further analysis.

### 3.4. Polarized Light Microscopic Observation of Emulsion Droplets and Size Measurement

The morphology of the droplets formed in the emulsion was examined by a polarized light microscope (Axioplan 2 imaging and Zeiss Axiophot 2 universal microscope, Carl Zeiss Inc., Jena, Germany). The images were taken with a Retiga 1300 camera linked to Northern Eclipse software. The distribution of the droplet sizes from the images was analyzed via ImageJ 1.x software.

### 3.5. Puree Sample Preparation

Puree samples (salmon, chicken, chicken stew, and beef) were stored at −20 °C and thawed to room temperature for the experiment. A portion of the puree sample was replaced at 5 and 10% with ultrasound-treated emulsion gels of 3% psyllium husk and 0–20% inulin. Each prepared sample was mixed until homogeneous using a Tissuemiser (Fisher Scientific, Ottawa, ON, Canada). After each mixing, the resultant puree samples were stored at −20 °C until analyzed.

### 3.6. Cryo-Scanning Electron Microscopic Observation of Emulsion Gels and Puree Samples

Emulsion gels and puree samples were observed under cryo-SEM (cryo-scanning electron microscope, Nano Imaging Facility Laboratory of Carleton University, Ottawa, ON, Canada). The method of Liu & Lanier (2015) was employed with slight modification [36]. The internal portion of the emulsion gels and puree samples was cut into blocks of 0.5 cm, and the blocks were then frozen on a metal plate surrounded by liquid nitrogen for 20 s. The frozen block was placed on a copper specimen holder and observed under a Cryo-SEM lens. The microscope was operated at 20 kV in low vacuum mode (40 Pa), with the temperature below −50 °C.

### 3.7. Total Expressible Fluid (TEF) Determination

The total expressible fluid (TEF) was determined according to the procedures described by Ismail et al. (2021) and Colmenero et al. (1995), with minor modifications [35,58]. Three replicates of control puree, 5% and 10% *w*/*w* emulsion prepared purees (~10 g) at room temperature were centrifuged (1 min, 3250× *g*), heated in a water bath (30 min, 70 °C), and immediately recentrifuged. For microwave oven heating, samples prepared as for water bath were centrifuged (1 min, 3250× *g*) and heated in a household microwave oven (Model DMW799BL, Danby, ON, Canada) for 1 min at 700 W and immediately centrifuged. The supernatant was removed, and the residue was weighed. The total expressible fluid (%) was calculated according to the following equation (Ismail et al., 2021) [58]:

(1) $$[\frac{Weight\;of\;the\;sample\;before\;heating-Weight\;of\;the\;sample\;after\;heating}{Weight\;of\;the\;sample\;before\;heating}]\times 100,$$

### 3.8. Texture Analysis

The firmness of samples was determined using the CT3 Texture Analyzer (Brookfield Engineering Labs Inc., Middleboro, MA, USA). The trigger force of the analysis was set to 5 g, and a cylindrical piston of 120 mm size was used to penetrate the sample at a depth of 10 mm at 2 mm /s speed. The results of the test are displayed as force (g). Tests for each type of sample were conducted in triplicate.

### 3.9. α-Amylase and α-Glucosidase Inhibitory Activities

The inhibitory activities of emulsion gels and puree samples against α-amylase and α-glucosidase were determined following standard methods with minor modifications [59].

For α-amylase inhibition, 20 μL α-amylase (1 U/mL) and 60 μL of diluted sample were preincubated in a glass tube at room temperature for 30 min. Then, 80 μL of 1% soluble starch in 20 mM phosphate buffer (pH 6.9) was added as substrate and incubated at 37 °C for 10 min, followed by the addition of 80 μL DNS reagent and boiling for 10 min. The absorbance was measured at 540 nm using a microplate reader (BioTek Cytation 5, Ottawa, ON, Canada).

For α-glucosidase inhibition, in a 96-well plate, 50 μL phosphate buffer (0.1 mM, pH 6.9), 10 μL α-glucosidase (1 U/mL), and 20 μL of diluted sample were preincubated at 37 °C for 15 min. Then, 20 μL p-NPG (1 mM) was added and incubated for 30 min at 37 °C. The reaction was stopped with 50 μL Na_2_CO_3_ (0.1 M), and absorbance was measured at 405 nm.

For both assays, emulsion gels (3% psyllium husk, 0–20% inulin) were diluted in buffer to 5 mg/mL, centrifuged (10,000 rpm, 2 min, room temperature), and the supernatant was used. Puree samples were diluted to 50 mg/mL, centrifuged under the same conditions, and the supernatant was used. Sample and enzyme blanks were prepared with 20 mM phosphate buffer (pH 6.9), replacing puree or enzyme, respectively.

Inhibitory activity (%) was calculated as:

[(Abs_blank_ − Abs_sample_)/Abs_blank_] × 100, (2)

where Abs_blank_ is the absorbance of the bank and Abs_sample_ is the absorbance of the sample.

### 3.10. International Dysphagia Diet Standardization Initiative (IDDSI) Tests

The International Dysphagia Diet Standardization Initiative (IDDSI) provides a framework for categorizing texturally modified food into eight levels, ranging from 0 to 7, and recommends a combination of tests to determine the appropriate level for a texture-modified food (IDDSI Framework testing methods 2.0/2019, 2019). A fork pressure test was conducted by pressing the puree samples onto a fork using the thumb. For the spoon tilt test, the purée samples were held on a spoon, and their behaviour was observed as the spoon was tilted to allow the sample to slide off.

### 3.11. Statistical Analysis

Experiments were conducted as a two-factor design with independently prepared batches per treatment in triplicate. Puree type and inulin concentration as factors were evaluated with ANOVA (SAS version 9.4). Duncan’s test (α = 0.05) was used to determine pairwise differences. Results are presented as mean ± SD; different letters denote significant difference.

## 4. Conclusions

In this study, an innovative allergy-free psyllium husk–inulin (up to 20%) emulsion gel was developed, in which the inulin molecule formed a protective layer on emulsion droplets. Ultrasonic homogenization further enhanced the emulsion stability by reducing the emulsion droplet size and improving the formation of an inulin layer to encapsulate the emulsion droplet. The inclusion of the husk–inulin emulsion gel in meat puree reduced the fluid release during water bath and microwave oven heating, and can delay the action of carbohydrate-hydrolyzing enzymes (α-amylase and α-glucosidase). Moreover, the addition of emulsion gels at 10% *w*/*w* substitution did not change the firmness of purees. The modified puree samples can be classified as IDDSI Level 4, which is suitable for people with dysphagia. This property makes it possible to use emulsion gels in food preparation for people with dysphagia. This study demonstrates that the gelling properties of psyllium husk can be used in forming emulsion gels with a positive impact on texturally modified food products. As such, the results present another ingredient that can be explored in emulsion-based foods.

For practical application, sensory and processing feasibility should be considered. Higher inulin (>20% *w*/*w*) can increase creaminess yet may shift flavour. Formulation should, therefore, be optimized with targeted sensory/tribology checks in the intended matrices. Industrial use of ultrasound is technically feasible via in-line systems, but temperature control must be balanced against throughput.

## Data Availability

Data presented in this study are available on request from the corresponding author.
